# Computed tomography-based radiomics to assess risk stratification in pediatric malignant peripheral neuroblastic tumors

**DOI:** 10.1097/MD.0000000000035690

**Published:** 2023-11-24

**Authors:** Xiaoxia Wang, Xinrong Wang, Tingfan Wu, Liwei Hu, Min Xu, Jingyan Tang, Xin Li, Yumin Zhong

**Affiliations:** a Department of Radiology, Shanghai Children’s Medical Center, Shanghai Jiao Tong University, School of Medicine, Shanghai, China; b General Electric China Co., Ltd, Shanghai, China; c Department of Surgery, Shanghai Children’s Medical Center, Shanghai Jiao Tong University, School of Medicine, Shanghai, China; d Department of Hematology and Oncology, Shanghai Children’s Medical Center, Shanghai Jiao Tong University, School of Medicine, Shanghai, China.

**Keywords:** children, neuroblastoma, peripheral neuroblastic tumors, radiomics, risk stratification

## Abstract

This study aimed to develop and validate an analysis system based on preoperative computed tomography (CT) to predict the risk stratification in pediatric malignant peripheral neuroblastic tumors (PNTs). A total of 405 patients with malignant PNTs (184 girls and 221 boys; mean age, 33.8 ± 29.1 months) were retrospectively evaluated between January 2010 and June 2018. Radiomic features were extracted from manually segmented tumors on preoperative CT images. Spearman’s rank correlation coefficient and the least absolute shrinkage and selection operator (LASSO) were used to eliminate redundancy and select features. A risk model was built to stratify low-, intermediate-, and high-risk groups. An image-defined risk factor (IDRFs) model was developed to classify 266 patients with malignant PNTs and one or more IDRFs into high-risk and non-high-risk groups. The performance of the predictive models was evaluated with respect to accuracy (Acc) and receiver operating characteristic (ROC) curve, including the area under the ROC curve (AUC). The risk model demonstrated good discrimination capability, with an area under the curve (AUC) of 0.903 to distinguish high-risk from non-high-risk groups, and 0.747 to classify intermediate- and low-risk groups. In the IDRF-based risk model with the number of IDRFs, the AUC was 0.876 for classifying the high-risk and non-high-risk groups. Radiomic analysis based on preoperative CT images has the potential to stratify the risk of pediatric malignant PNTs. It had outstanding efficiency in distinguishing patients in the high-risk group, and this predictive model of risk stratification could assist in selecting optimal aggressive treatment options.

## 1. Introduction

Peripheral neuroblastic tumors (PNTs) are highly heterogeneous. They arise in primitive neuroblasts derived from the embryonic neural crest, and can occur anywhere within the sympathetic nervous system. Malignant PNTs, including neuroblastoma and ganglioneuroblastoma, are the most common malignant extracranial solid tumors that occur during infancy and childhood.^[1]^ They account for approximately 8% of childhood malignancies but are attributed to approximately 12% of cancer deaths in children younger than 15 years of age.^[2,3]^

The prognosis of malignant PNTs varies significantly based on patient age, disease stage, and biological factors, including the histological category and grade of tumor differentiation, amplification of the *MYCN* oncogene, DNA ploidy, and chromosomal aberrations. Based on the above factors, the International Neuroblastoma Risk Group (INRG) pretreatment risk stratification schema classifies malignant PNTs into low-, intermediate-, and high-risk groups.^[4–6]^ Treatment of childhood PNTs is stratified according to risk groups and can range from surgery alone for low-risk tumors to aggressive multimodal therapy, including surgical resection, chemotherapy, high-dose chemotherapy with autologous stem cell transplantation, radiotherapy, and immunotherapy for high-risk tumors. Patients with malignant PNTs require comprehensive assessment, including medical imaging, biological marker detection, tumor surgery or biopsy, and bone marrow biopsy, for risk stratification before treatment.^[7,8]^ Except for imaging evaluation of the INRG stage, other methods for the evaluation of risk factors are invasive and take longer or require more advanced laboratory equipment and technology to obtain evaluable data. Therefore, it may be difficult for physicians in most grassroots hospitals in underdeveloped areas to evaluate risk stratification comprehensively. Thus, a rapid, easy, and noninvasive method to pre-therapeutically stratify risks will be useful for the assessment and selection of treatment protocols for pediatric malignant PNTs.

Radiomics is a new noninvasive method that can convert captured images into high-throughput quantitative data and has been successfully applied to cancer research.^[9,10]^ In contrast to conventional visual interpretation, various studies have shown that radiomics features can provide insightful quantitative information regarding intratumoral heterogeneity and microenvironment interactions.^[11–13]^ Radiomics has demonstrated its potential in identifying the risk stratification and prognosis of malignant tumors.^[14–16]^ Nevertheless, there has been limited research on the development of noninvasive CT-based radiomics models to predict risk stratification in malignant PNTs before treatment. Therefore, we hypothesized that a preoperative radiomics analysis system based on features extracted from CT images could assist in risk stratification of malignant PNTs.

In this study, we collected CT images and clinical data that were used to develop radiomic prediction models to stratify risk before treatment of pediatric malignant PNTs. We also explored the textural parameters and clinical features of pediatric malignant PNTs that could affect the accuracy of risk stratification using this radiomics-based system.

## 2. Methods

This study was approved by the Research Ethics Committee of Shanghai Children’s Medical Centre and the Regulatory and Institutional Review Committee. As this was a retrospective study, the requirement for informed consent from the patients was waived.

### 2.1. Patient and data collection

**Figure 1.**
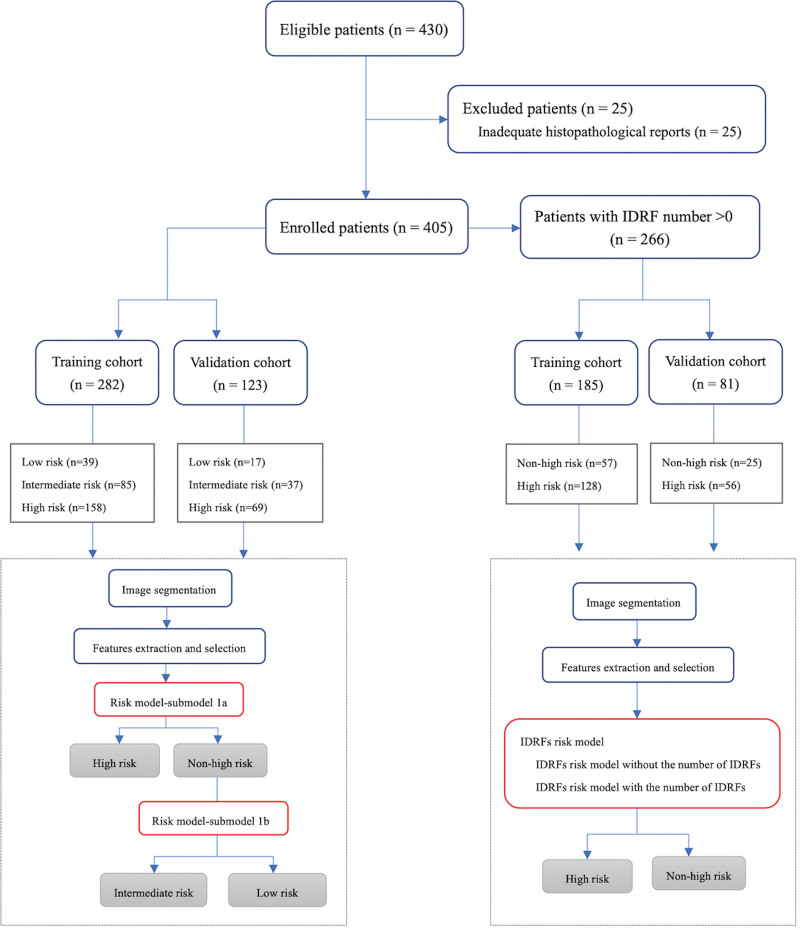
The flowchart of the proposed radiomics system.

We retrospectively screened pediatric patients with malignant PNTs from our hospital’s medical record database between January 2010 and June 2018. The inclusion criteria were as follows: initial case with a pathologically confirmed diagnosis, including neuroblastoma and ganglioneuroblastoma, by biopsy or resection of the tumor; post-contrast-enhanced CT for tumor imaging less than 1 month before surgery; available preoperative CT images; and complete clinical data for comprehensive assessment of risk stratification. The exclusion criteria were as follows: inadequate histopathological reports and previous tumor treatment including chemotherapy, radiotherapy, or surgical treatment. Consequently, 25 patients were excluded owing to inadequate histopathological reports, and 405 patients (184 girls and 221 boys; mean age, 33.8 ± 29.1 [standard deviation] months) were enrolled (Fig. 1).

In this study, a retrospective evaluation of the image-defined risk factors (IDRFs) of primary tumors and tumor stage was performed according to the INRG staging system. IDRFs of pediatric malignant PNTs were defined as features detected on imaging that existed when the tumor encased or invaded vital structures, most commonly the major blood vessels (Tables S1 and S2, Supplemental Digital Content, http://links.lww.com/MD/K446, http://links.lww.com/MD/K447).^[5]^ These imaging findings were performed by a single pediatric radiologist with more than 10 years of experience, and the number of IDRFs in each case was recorded in 266 patients who presented with one or more IDRFs.

The clinical characteristics of 405 patients, including age at diagnosis, sex, tumor site, and risk group, were obtained from medical records. Of the 405 patients, 56, 122, and 227 were classified into low-, intermediate-, and high-risk groups, respectively, according to the INRG pretreatment classification (Table S3, Supplemental Digital Content, http://links.lww.com/MD/K448).^[8]^ Of 266 patients with one or more IDRFs, 13 were in the low-risk group, 69 in the intermediate-risk group, and 184 in the high-risk group.

### 2.2. CT image acquisition

We used preoperative and post-contrast-enhanced CT Digital Imaging and Communications in Medicine (DICOM) data from the Picture Archiving and Communication System. All CT images were collected using a 16-row CT scanner (Lightspeed 16, General Electric Medical Systems), 64-row CT scanner (Discovery HD 750, General Electric Medical Systems), and 320-row CT scanner (Aquilion ONE, Canon Medical Systems). The numbers of patients who underwent scanning with the 3 CT scanners were 202, 172, and 31, respectively. Scanning parameters for the 16-row CT scanner were as follows: tube voltage, 120 Kv; tube current, 100 mA; slice thickness, 10 mm; rotation time, 0.5 s; pitch, 1.375; reconstruction slice thickness, 1.5 mm. The scanning parameters for the 64-row CT scanner were as follows: tube voltage, 80 Kv; automatic tube current; FOV (field of view (FOV), 32 mm; slice thickness, 10 mm; rotation time, 0.35 s; pitch, 1.375; reconstruction slice thickness,1.25 mm. The scanning parameters for the 320-row CT scanner were as follows: tube voltage, 80 Kv; automatic tube current, field of view (FOV), 24 mm; slice thickness, 10 mm; rotation time, 0.275 s; pitch, 1.375; reconstruction slice thickness 1.0 mm. The enhanced phase used a bolus injection of 2 mL/kg of iodinated contrast agent. Time of scan of delay, arterial phase, 18 to 25 s, portal venous phase, 35 to 45 s.

CT images from different manufacturers have different signal intensities; however, the radiomics features of the same brand of CT scanner differ less when the scan parameters are constant. To reduce the impact of slice thickness and bin width on feature invariance, we performed resampling before feature extraction and employed the optimization of gray-level discretization to potentially improve the predictive value.^[17]^

### 2.3. Image segmentation, radiomics features extraction, and selection

The region of interest was defined as the location of the primary tumor, and all segmentations of 405 tumors were manually delineated by 7 radiographers who were rigorously trained on all pretreatment arterial phases of CT images using ITK-SNAP software version 2.2.0 (www.itksnap.org). The segmentation results were checked and unified by an experienced pediatric radiologist with more than 10 years of experience. All the involved clinicians were blinded to the clinical data of the enrolled patients. After tumor segmentation, radiomic features were extracted from the manually segmented tumors using an I.F. (Intelligence Foundry 2.1, GE Healthcare). Features were derived from original features, including first-order histogram, tumor shape, gray-level co-occurrence matrix, gray-level run length matrix, gray-level size zone matrix, neighboring gray-tone difference matrix, gray-level dependence matrix, and filter-based methods including the wavelet transform-local binary pattern and local binary pattern with pyramid representation. Furthermore, to obtain the most significant features, Spearman’s rank correlation coefficient with 0.9 threshold and least absolute shrinkage and selection operator were used to eliminate redundancy and select features, respectively.

### 2.4. Construction and validation of the prediction models

Of the entire data cohort comprising 405 patients, 70% (n = 282) were enrolled in the training cohort to develop a prediction model. To obtain an interpretable model, we used a hierarchical multilabel classification based on the findings of Vens et al and Gibaja et al^[18,19]^ To build a classifier (risk model) for the risk stratification of the 3 groups, 2 logistic regression submodels were developed based on the HCM. Patients were first entered into sub-model 1a for high-risk group identification. If they were diagnosed as belonging to the non-high-risk group, they were entered into submodel 1b for differential diagnosis of either the intermediate-risk or low-risk groups. The INRG staging system, which is an important prognostic factor for risk stratification, includes 2 stages of localized disease (L1 and L2) that are dependent on the presence or absence of IDRFs.^[5]^ Besides, IDRFs of pediatric malignant PNTs were useful in predicting surgical risk and completeness of tumor resection, as well as significant indicators of prognosis.^[20–22]^ Among malignant PNTs patients with one or more IDRFs, it is difficult to distinguish the high-risk group from the non-high-risk group through medical imaging. Therefore, we developed a predictive model (IDRFs risk model), with or without the number of IDRFs, to identify high-risk and non-high-risk groups based on 266 patients who presented with one or more IDRFs, of which 185 were enrolled in the training cohort.

Validation cohorts were used to assess the predictive ability of selected radiomics features. Based on the prediction results of the validation cohort, we used accuracy (Acc) and evaluation indicators related to the receiver operating characteristic (ROC) curve, including the area under the ROC curve (AUC), sensitivity, and specificity, to evaluate the performance of the prediction models. The workflow of the proposed radiomics system is illustrated in Figure 1.

### 2.5. Statistical analysis

All analyses were performed using I.F. (Intelligence Foundry 2.1, GE Healthcare). Statistical analysis of clinical information was performed using SPSS (version 25.0, SPSS, Inc., Chicago, IL). Univariate analysis was used to compare the differences in the clinical factors between the 2 groups using the chi-square test or Fisher’s exact test for categorical variables, and the Mann–Whitney *U* test for continuous variables. A two-tailed test was used, and *P *< .05.

## 3. Results

### 3.1. Clinical characteristics

Patient characteristics in the training and validation cohorts of the 2 models (risk model and IDRFs risk model) are summarized in Table 1. In the risk model, there were no significant differences in the sample distribution between the training and validation cohorts (*P* = .593 for submodel 1a, *P* = .415 for submodel 1b). The density distributions of the samples in both cohorts are consistent (Figure S1A and B, Supplemental Digital Content, http://links.lww.com/MD/K450), which justified their use as the training and validation cohorts. In the IDRFs risk model, the density distribution of the samples in the 2 cohorts was consistent (Figure S1C, Supplemental Digital Content, http://links.lww.com/MD/K450).

**Table 1:** Characteristics of patients in the training and validation cohorts.

Characteristic	Risk model				IDRFs risk model (n = 266)	
	Submodel 1a (n = 405)		Submodel 1b (n = 178)		Training cohort (n = 185)	Validation cohort (n = 81)
	Training cohort (n = 282)	Validation cohort (n = 123)	Training cohort (n = 124)	Validation cohort (n = 54)		
Age, mean ± SD, months	34.6 ± 28.9	21.5 ± 24.9	32.0 ± 29.9	20.2 ± 24.2	36.7 ± 29.1	38.5 ± 32.7
Sex, N (%)						
Male	155 (55.0)	67 (54.0)	75 (61.0)	33 (61.1)	102 (55.1)	49 (60.5)
Female	127 (45.0)	57 (46.0)	48 (39.0)	21 (38.9)	83 (44.9)	32 (39.5)
Tumor sites						
Neck	12 (4.2)	6 (4.9)	4 (3.2)	2 (3.7)	10 (5.4)	5 (6.2)
Chest	53 (18.8)	34 (27.6)	32 (25.8)	17 (31.5)	27 (14.6)	13 (16.0)
Abdomen	203 (72.0)	77 (62.6)	84 (67.7)	33 (61.1)	145 (78.4)	61 (75.3)
Pelvis	14 (5.0)	6 (4.9)	4 (3.2)	2 (3.7)	3 (1.6)	2 (2.5)
Risk groups, N (%)						
Low		39 (31.5)		17 (31.5)	9 (4.9)	4 (4.9)
Intermediate		85 (68.6)		37 (68.5)	48 (25.9)	21 (25.9)
Low and Intermediate	124 (44.0)		54 (43.9)		57 (30.8)	25 (30.9)
High	158 (56.0)		69 (56.1)		128 (61.2)	56 (49.1)
Mean number of IDRFs						
Low and Intermediate					2.0 ± 1.2	2.2 ± 2.4
High					3.7 ± 2.2	3.8 ± 1.9

IDRFs = image-defined risk factors.

### 3.2. Radiomics features

**Figure 2.**
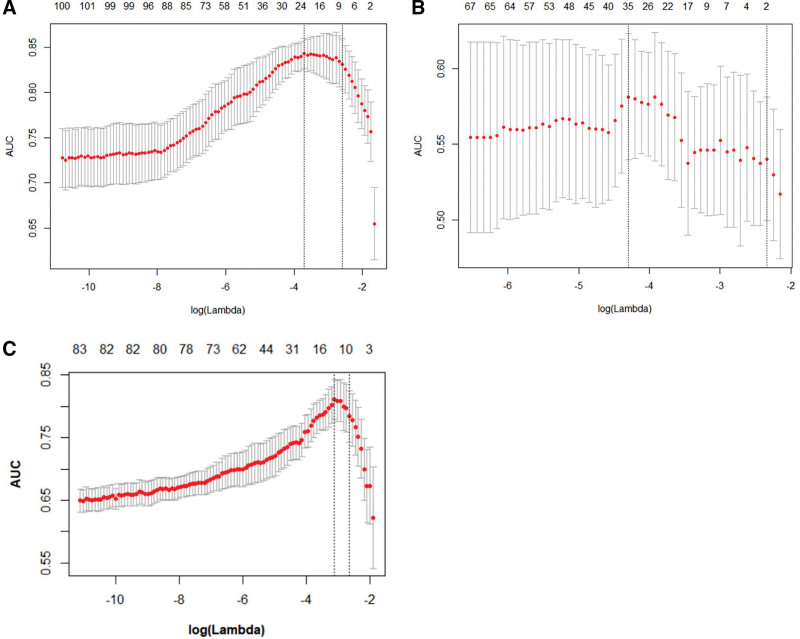
Feature selection using the least absolute shrinkage and selection operator (LASSO) binary logistic regression model in the risk model [submodel 1a for classifying high- and non-high-risk groups (A), submodel 1b for classifying low- and intermediate -risk groups (B)] and IDRFs risk model for classifying high- and non-high-risk group (C). The area under the receiver operating characteristic (AUC) curve was plotted versus log (Lambda). The AUC index of the training cohort was used as the evaluation index for LASSO equation. Along with the increase of lambda value, the number of remaining features had decreased. In submodel 1a (A), the AUC reached a maximum value of 0.84 when the number of features was 23, then the remaining 23 features were selected as the result of LASSO dimensionality reduced. In submodel 1b (B), the AUC reached the maximum value of 0.75 when the remaining 35 features were selected as the result of LASSO dimensionality reduction.

**Figure 3.**
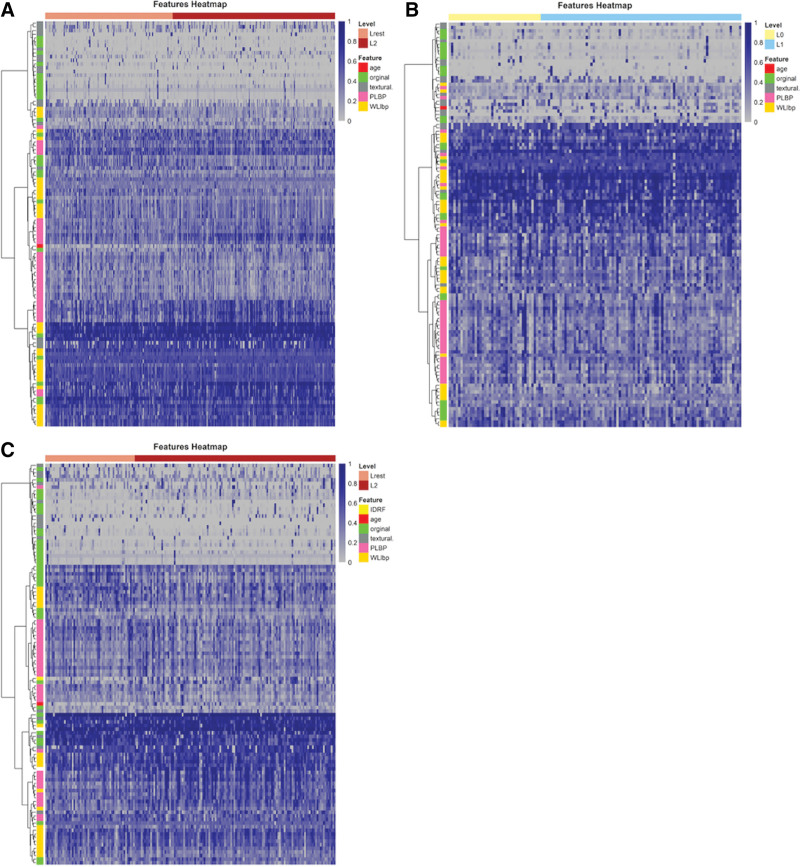
Heat map showed the clustering results of all features in the training cohort for risk model [submodel 1a (A), submodel 1b (B)] and IDRFs-Risk-Model (C). Radiomics features (original, textural, PLBP, WLlbp) were clustered into feature trees according to eigenvalue distances. (A, C) Hierarchical clustering analysis of features in submodel 1a (A) and IDRFs risk model (C). It showed that eigenvalues of the non-high-risk group level (Lelse) and high-risk group level (L2) were different in the pink (PLPB) feature domain. The color of PLPB in the high-risk group was darker than that in the non-high-risk group, indicating that PLPB features of the high-risk group was closer to 1, and the non-high-risk group was closer to 0. (B) Hierarchical clustering analysis of features in submodel 1b. There were no significant differences the feature trees between L0 level (low-risk group) and L1 level (intermediate-risk group).

**Figure 4.**
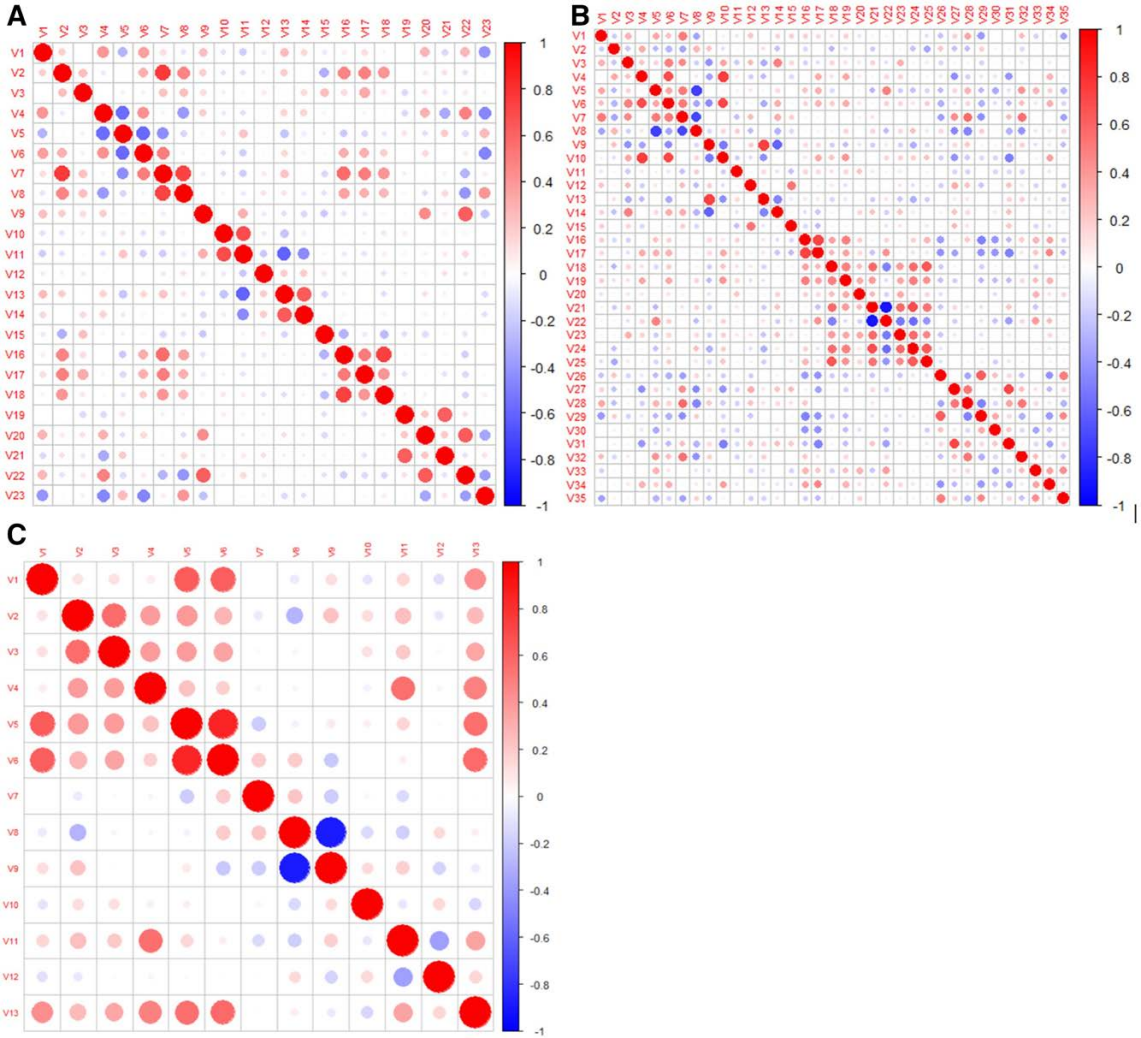
The Spearman correlation coefficient between features of Risk model [submodel 1a (A), submodel 1b (B)] and of IDRFs risk model (C), respectively. IDRFs = image-defined risk factors.

**Figure 5.**
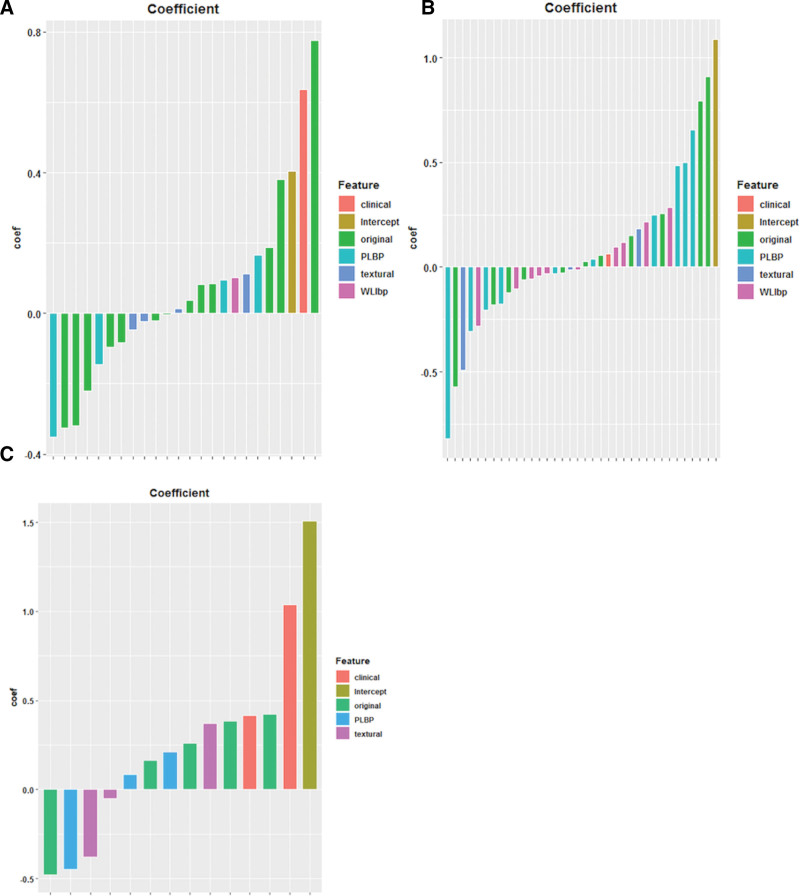
Visualization of feature coefficient for risk model [submodel 1a (A), submodel 1b (B)] and IDRFs risk model (C). Apart from the fact that Intercept was a constant of the equation, the same color represented a class of features. The pink feature comprised clinical features including age at diagnosis in submodel 1a and submodel 1b, and age at diagnosis and the number of IDRFs in the IDRFs risk model. In submodel 1a (A), the maximum absolute value of the coefficient in original features was about 0.78, while that in clinical features (age at diagnosis) and PLBP features was about 0.64 and 0.35, respectively. In submodel 1b (B), the maximum absolute value of the coefficient in original, PLBP, and textural features was about 0.91, 0.82, and 0.49, respectively. In the IDRFs risk model (C), the maximum absolute value of the coefficient in clinical features, original, and PLBP features was 1.03, 0.48, and 0.45, respectively. IDRFs = image-defined risk factors, PLBP = local binary pattern with pyramid representation.

A total of 263 radiomic features were extracted from the segmented tumor regions. After feature selection, 23 and 35 features including one clinical feature (age at diagnosis), were selected for submodels 1a and 1b, respectively (Fig. 2A and B). For the IDRFs risk model (Fig. 2C), 11 radiomic features were selected and 2 clinical features (age at diagnosis and number of IDRFs) were added to the model. Details of the feature coefficients for the risk model and IDRFs risk model are presented in Table S4 (Supplemental Digital Content, http://links.lww.com/MD/K449). The clustering results for all the features are shown in Figure 3. The PLPB features were important in distinguishing between the high-risk and non-high-risk groups in submodel 1a (Fig. 3A) and IDRFs risk model (Fig. 3C). In submodel 1b (Fig. 3B), there were no significant differences in the features between the low- and intermediate-risk groups. Spearman’s correlation of the remaining features was low in both submodels 1a and 1b, and in the IDRFs risk model (Fig. 4). The feature coefficients for the risk model are shown in Figure 5A and B, and the original features accounted for the highest proportion in both sub-models 1a and 1b. Clinical features (age at diagnosis and number of IDRFs) accounted for the highest proportion in the IDRF risk model, followed by the original and local binary pattern with pyramid representation features (Fig. 5C).

### 3.3. Prediction model

**Figure 6.**
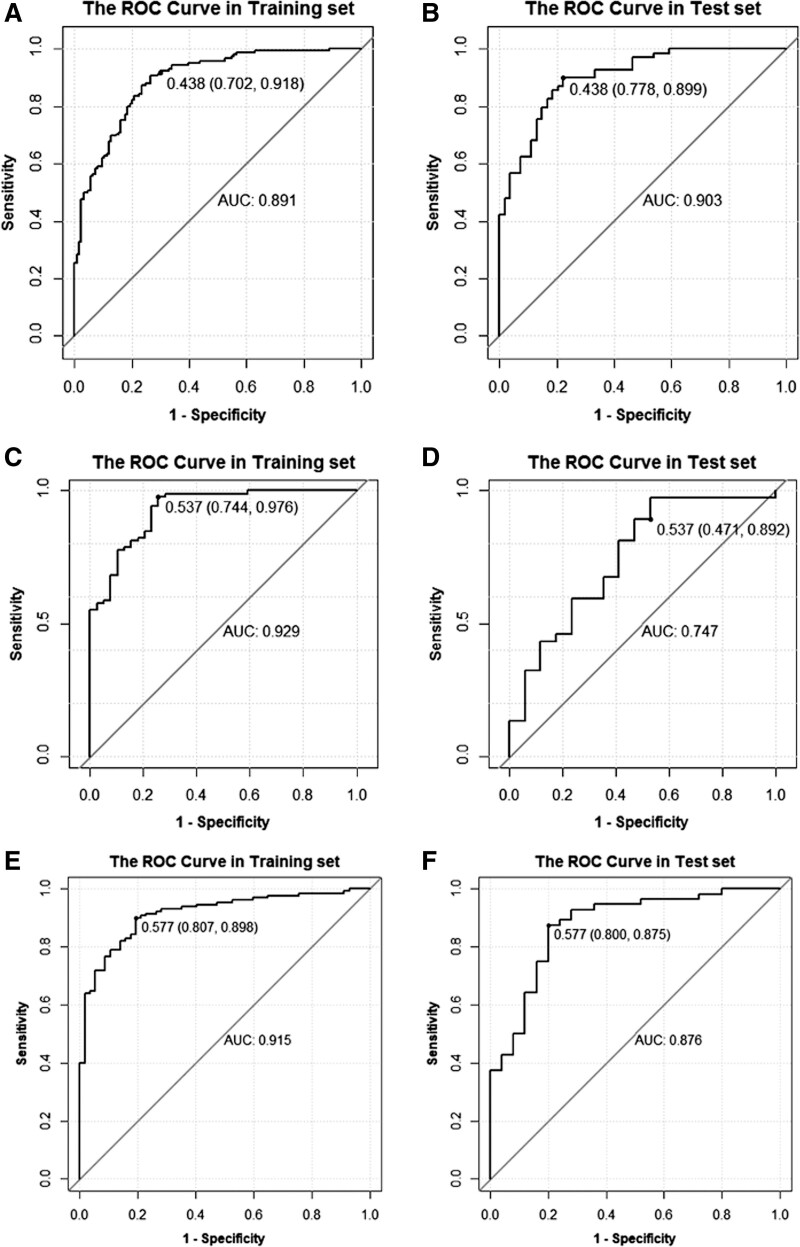
Receiver operating characteristic (ROC) curves of the radiomics models for the training cohort (A) and validation cohort (B) in submodel 1a; the training cohort (C) and validation cohort (D) in submodel 1b; and the training cohort (E) and validation cohort (F) in the IDRFs risk model with the number of IDRFs. IDRFs = image-defined risk factors.

The models demonstrated good discrimination in both training and validation cohorts. The diagnostic metrics of the validation cohort are summarized in Table 2. The ROC curves for the radiomics models are shown in Figure 6. In the validation cohort of the risk model, the AUC values were 0.903 for submodel 1a and 0.747 for submodel 1b. In the validation cohort of the IDRFs risk model with the number of IDRFs, the AUC, Acc, sensitivity, and specificity values were 0.876, 85.2%, 87.5%, and 80.0% and 0.864, 81.5%, 80.4%, and 84.0%, respectively.

**Table 2:** Diagnostic metrics of the risk model and IDRFs risk model in the validation cohort.

Model name		AUC	Accuracy	Sensitivity	Specificity
Risk model	Submodel 1a	0.903	84.6%	89.9%	77.8%
	Submodel 1b	0.747	75.9%	89.2%	47.1%
IDRFs risk model	With the number of IDRFs	0.876	85.2%	87.5%	80.0%
	Without the number of IDRFs	0.864	81.5%	80.4%	84.0%

AUC = area under the receiver operating characteristics curve, IDRFs = image-defined risk factors.

## 4. Discussion

In the present study, 405 children with malignant PNTs were enrolled, and 2 risk stratification models, the risk model and IDRFs risk model based on pretreatment CT images, were developed. Our results proved valuable in stratifying and distinguishing high-risk groups with AUCs of 0.903 and 0.876, respectively, and seemed suitable for clinical application. In recent years, several radiomics studies of malignant PNTs have reported features associated with the prediction of tumor histology,^[23]^ MYCN amplification status,^[24–26]^ bone marrow involvement,^[27]^ response to neoadjuvant chemotherapy,^[28]^ recurrence,^[29]^ prognosis,^[30]^ and differentiation between high-risk and non-high-risk groups.^[31]^ Nearly half of the radiomics studies were based on ^18^F-fluorodeoxyglucose positron emission tomography/computed tomography (^18^F-FDG PET/CT). Feng et al^[31]^ reported that the AUCs of the radiomics model for distinguishing high- and non-high-risk patients in the training and validation sets were 0.957 and 0.933, respectively, which were higher than those in our study. The main reason for this may be that 18F-FDG PET/CT is an integrated imaging modality of PET and CT that can provide both metabolic and anatomical information.^[32]^

In total, 405 patients were included in the risk model. Twenty-three features were selected for submodel 1a and 35 features were selected for submodel 1b. The total number of cases analyzed was appropriate because more than 4 patients/feature were included.^[33]^ These radiomics features provide more information regarding textural characteristics and heterogeneity within a tumor. Additionally, the age of the patients at diagnosis is an important clinical feature for classifying high-risk and non-high-risk groups. It is an important risk factor for risk stratification in malignant PNTs and is considered an alternative for underlying biological characteristics because younger children are more likely to have tumors with biological features that are associated with a benign clinical course. In contrast, older age was a prognostic factor for poor survival.^[3,34]^ By comparing submodel 1a and submodel 1b, patients’ age at diagnosis in submodel 1a was older than that in submodel 1b (training cohort:34.59 ± 28.86 vs 21.50 ± 24.94 and validation cohort:32.04 ± 29.91 vs 20.24 ± 24.21, respectively). Therefore, age at diagnosis was selected as the most promising feature for the stratification of high-risk patients.

The radiomics score demonstrated outstanding discrimination in the validation cohort (AUC, 0.903; Acc, 84.6% for submodel 1a; AUC, 0.747; Acc, 75.9% for submodel 1b). The AUC and Acc values for both submodels demonstrated that the prediction models based on the radiomics method yielded high performance in the preoperative prediction of risk stratification for pediatric malignant PNTs, especially in submodel 1a, by simplifying the classification by pediatric oncologists between the high-risk and non-high-risk groups. Prediction submodel 1a for the identification of high-risk and non-high-risk groups had a higher AUC and Acc than submodel 1b (classifying intermediate- and low-risk groups), suggesting that submodel 1a had a better ability to distinguish high-risk groups, and provided a reference for a closer follow-up plan for patients predicted to be in the high-risk group. Most patients in the overall cohort (n = 405) belonged to the high-risk group (n = 227). The imbalanced ratios of patients with malignant PNTs and a small patient pool in both the low- and intermediate-risk groups in submodel 1b may be the main reason for the less reliable outcomes than those in submodel 1a. Moreover, the specificity of submodel 1a was significantly higher than that of submodel 1b (0.778 vs 0.471), indicating that it could efficiently distinguish high-risk from non-high-risk groups. These results likely indicate that radiomics features provide more information regarding heterogeneity within malignant PNTs in the high-risk group, such that the radiomics signature can successfully distinguish patients in the high-risk group from those in submodel 1a. At the same time, the difference in radiomics features between the low- and intermediate-risk groups was smaller and likely led to lower specificity for submodel 1b.

IDRFs was defined according to the International Neuroblastoma Risk Group Project.^[5]^ IDRFs of pediatric malignant PNTs are features detected on imaging that are mostly recommended for use as predictors of surgical risk and completeness of tumor resection.^[20,21]^ The latest study by Zhang et al reported that the presence of ≥ 4 IDRFs was useful for predicting surgical outcomes and significant indicators of poor prognosis.^[22]^ Their study also revealed a highly significant negative correlation between the number of IDRFs and the possibility of complete neuroblastoma removal. In our study, the IDRFs risk model with a number of IDRFs proved highly effective and showed promising results, with an AUC of 0.876 and an Acc of 85.2%, both of which were slightly higher than those of the model without IDRFs (AUC = 0.864; Acc = 81.5%). Thus, the IDRFs risk model verified the efficiency of risk stratification prediction for childhood malignant PNTs by employing clinical features and number of IDRFs combined with radiomic features. Moreover, the mean number of IDRFs was significantly higher in the high-risk group than that in the non-high-risk group. These results strongly verified that the number of IDRFs is an effective indicator for risk stratification of malignant PNTs, before proving that the extraction of features based on CT images combined with the number of IDRFs could be applied in risk stratification for high-risk and non-high-risk groups of pediatric malignant PNTs.

Our study had some limitations. First, the data used were derived from only one medical institution, and although our study cohort was relatively large, the established risk-stratification model should be refined and validated using data from a multicenter patient pool. Second, the samples in sub-model 1b were inadequate because the number of cases in the low-risk group was naturally low, which likely made it difficult for the classifier to fit into the model. Therefore, research on a larger cohort is needed to fully understand the radiomic features of patients in the low-risk groups. It may then be possible to establish a more efficient model in the future to significantly contribute to and support differentiation between low- and intermediate-risk groups when diagnosing malignant pediatric PNTs. Third, the radiomic features of tumors at different primary sites could differ, which could affect the accuracy of the risk model for pediatric malignant PNTs. However, in our study, most patients had abdominal tumors, whereas tumors in the neck or pelvis were rare. An imbalanced ratio of malignant PNTs at different sites and a small patient pool with neck or pelvic tumors may have affected the reliability of the radiomic features. Therefore, we plan to continue to collect data from patients with malignant PNTs at various sites.

## 5. Conclusions

In conclusion, our results suggest that a radiomics system based on preoperative CT images can potentially stratify the risks. It showed outstanding efficiency in distinguishing high-risk patients who were expected to exhibit poor prognosis. This would allow the selection of aggressive chemotherapy treatments to improve the long-term survival of this group of patients.

## Acknowledgments

The authors would like to thank Ting Zuo, Jinye Lu, Jiayun Jiang, Qi Zhang, Haocheng Pan, and Shengyu Zhou for their contribution to image segmentation.

## Author contributions

**Conceptualization:** Xiaoxia Wang.

**Data curation:** Liwei Hu.

**Formal analysis:** Tingfan Wu.

**Investigation:** Xiaoxia Wang.

**Methodology:** Xiaoxia Wang.

**Project administration:** Yumin Zhong.

**Resources:** Min Xu, Jingyan Tang.

**Software:** Xinrong Wang, Tingfan Wu.

**Supervision:** Xin Li.

**Validation:** Xinrong Wang.

**Visualization:** Liwei Hu.

**Writing – original draft:** Xiaoxia Wang.

**Writing – review & editing:** Yumin Zhong.

## Supplementary Material
