# Supplementary material for: Computed tomography-based radiomics to assess risk stratification in pediatric malignant peripheral neuroblastic tumors
Source: Medicine (Baltimore). 2023 Nov 24;102(47):e35690. doi: 10.1097/MD.0000000000035690 (PMC10681616; doi:10.1097/MD.0000000000035690)
Supplement: Supplementary file 1 [file medi-102-e35690-s001.docx]

Article title: Computed tomography-based radiomics to assess risk stratification in pediatric malignant peripheral neuroblastic tumors

First author: Xiaoxia Wang

**Table S1**. Image-defined risk factors (IDRFs) in Neuroblastoma

| Tumor site | Description |
| --- | --- |
| Multiple body compartments | Ipsilateral tumor extension within two body compartments (neck and chest, chest and abdomen, abdomen and pelvis) |
| Neck | Tumor encasing carotid artery, vertebral artery, and/or internal jugular vein  Tumor extending to skull base  Tumor compressing trachea |
| Cervico-thoracic junction | Tumor encasing brachial plexus roots  Tumor encasing subclavian vessels, vertebral artery, and/or carotid artery  Tumor compressing trachea |
| Thorax | Tumor encasing aorta and/or major branches  Tumor compressing trachea and/or principal bronchi  Lower mediastinal tumor infiltrating costovertebral junction between T9 and T12 vertebral levels |
| Thoraco-abdominal junction | Tumor encasing aorta and/or vena cava |
| Abdomen/pelvis | Tumor infiltrating porta hepatis and/or hepatoduodenal ligament  Tumor encasing branches of superior mesenteric artery at mesenteric root  Tumor encasing origin of celiac axis and/or origin of superior mesenteric artery  Tumor invading one or both renal pedicles  Tumor encasing aorta and/or vena cava  Tumor encasing iliac vessels  Pelvic tumor crossing sciatic notch |
| Intraspinal tumor extension | Intraspinal tumor extension (whatever the location) provided that more than one-third of spinal canal in axial plane is invaded, the perimedullary leptomeningeal spaces are not visible, or the spinal cord signal intensity is abnormal |
| Infiltration of adjacent organs and structures | Pericardium, diaphragm, kidney, liver, duodenopancreatic block, and mesentery |
