# Supplementary material for: Computed tomography-based radiomics to assess risk stratification in pediatric malignant peripheral neuroblastic tumors
Source: Medicine (Baltimore). 2023 Nov 24;102(47):e35690. doi: 10.1097/MD.0000000000035690 (PMC10681616; doi:10.1097/MD.0000000000035690)
Supplement: Supplementary file 2 [file medi-102-e35690-s002.docx]

Article title: Computed tomography-based radiomics to assess risk stratification in pediatric malignant peripheral neuroblastic tumors

First author: Xiaoxia Wang

**Table S2.** Classification of International Neuroblastoma Risk Group Staging System

| Stage | Description |
| --- | --- |
| L1 | Localized tumor not involving vital structures as defined by the list of image-defined risk factors and confined to one body compartment. |
| L2 | Loco-regional tumor with presence of one or more image-defined risk factors. |
| M | Distant metastatic disease (except stage MS). |
| MS | Metastatic disease in children younger than 18 months with metastases confined to skin, liver, and/or bone marrow. |
