# Supplementary material for: Computed tomography-based radiomics to assess risk stratification in pediatric malignant peripheral neuroblastic tumors
Source: Medicine (Baltimore). 2023 Nov 24;102(47):e35690. doi: 10.1097/MD.0000000000035690 (PMC10681616; doi:10.1097/MD.0000000000035690)
Supplement: Supplementary file 3 [file medi-102-e35690-s003.docx]

Article title: Computed tomography-based radiomics to assess risk stratification in pediatric malignant peripheral neuroblastic tumors

First author: Xiaoxia Wang

**Table S3.** International Neuroblastoma Risk Group Pretreatment Classification Schema

| **Risk Group** | **INRG Stage** | **Age (months)** | **Histologic Category** | **Grade of Tumor Differentiation** | ***MYCN*** | **11q Aberration** | **DNA Ploidy** |
| --- | --- | --- | --- | --- | --- | --- | --- |
| Low | L1/L2 |  | GN maturing; GNB Intermixed |  |  |  |  |
|  | L1 |  | Any, except GN maturing or GNB Intermixed |  | NA |  |  |
|  | L2 | <18 | Any, except GN maturing or GNB Intermixed |  | NA | No |  |
|  |  | ≥18 | GNB nodular; neuroblastoma | Differentiating | NA | No |  |
|  | M | <18 |  |  | NA |  | Hyperdiploid |
|  | MS | <18 |  |  | NA | No |  |
| Intermediate | L2 | <18 | Any, except GN maturing or GNB Intermixed |  | NA | Yes |  |
|  |  | ≥18 | GNB nodular; neuroblastoma | Differentiating | NA | Yes |  |
|  |  |  |  | Poorly differentiated or undifferentiated |  |  |  |
|  | M | <18 |  |  | NA |  | Diploid |
| High | L1 |  | Any, except GN maturing or GNB Intermixed |  | Amp |  |  |
|  | L2 | ≥18 | GNB nodular; neuroblastoma |  | Amp |  |  |
|  | M | <18 |  |  | Amp |  |  |
|  |  | ≥18 |  |  |  |  |  |
|  | MS | <18 |  |  | NA | Yes |  |
|  |  | <18 |  |  | Amp |  |  |
