# Supplementary figures and images for: Computed tomography-based radiomics to assess risk stratification in pediatric malignant peripheral neuroblastic tumors
Source: Medicine (Baltimore). 2023 Nov 24;102(47):e35690. doi: 10.1097/MD.0000000000035690 (PMC10681616; doi:10.1097/MD.0000000000035690)

A
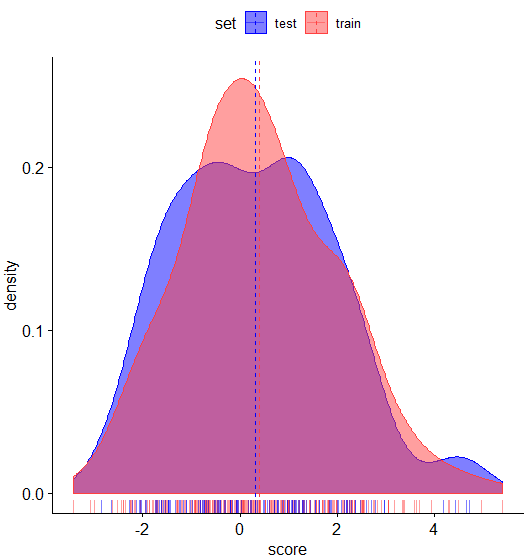


B
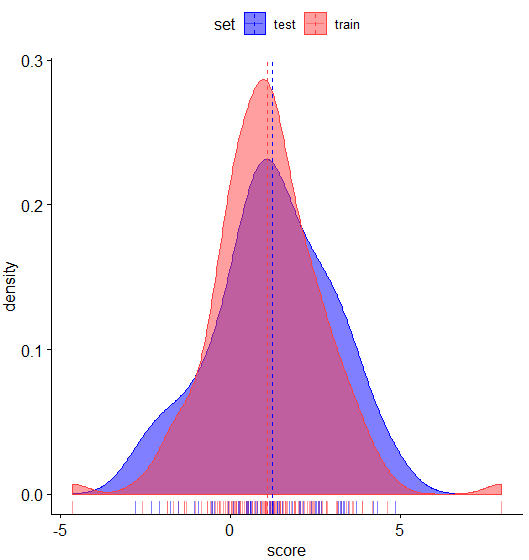


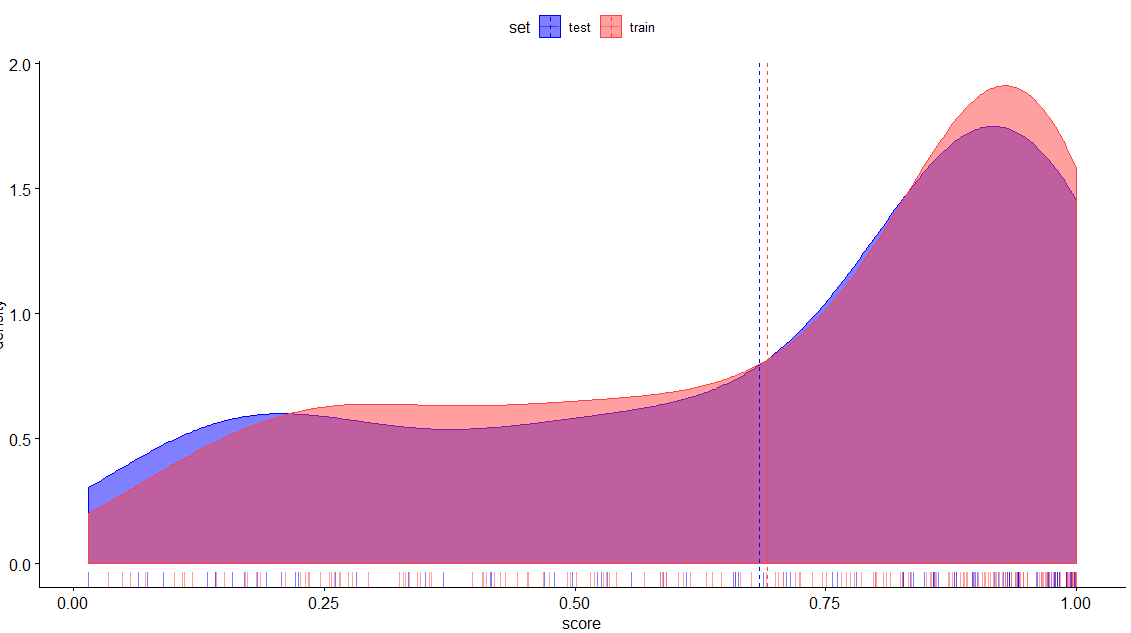


C

Supplement: Supplementary file 4 [file medi-102-e35690-s004.docx]
