# Supplementary material for: Computed tomography-based radiomics to assess risk stratification in pediatric malignant peripheral neuroblastic tumors
Source: Medicine (Baltimore). 2023 Nov 24;102(47):e35690. doi: 10.1097/MD.0000000000035690 (PMC10681616; doi:10.1097/MD.0000000000035690)
Supplement: Supplementary file 5 [file medi-102-e35690-s005.docx]

Article title: Computed tomography-based radiomics to assess risk stratification in pediatric malignant peripheral neuroblastic tumors

First author: Xiaoxia Wang

**Table 4S.** Feature coefficient for the Risk model and IDRFs risk model

| Risk model | | | | | | IDRFs risk model | | |
| --- | --- | --- | --- | --- | --- | --- | --- | --- |
| Submodel 1a | | | Submodel 1b | | |  |  |  |
| Number | Features | coefficient | Number | Features | coefficient | number | Features | coefficient |
| 1 | age | 0.636236848 | 1 | age | 0.06335245 | 1 | IDRF | 0.41776 |
| 2 | original_firstorder_Kurtosis | -0.09538248 | 2 | original_firstorder_10Percentile | -0.17852134 | 2 | age | 1.03658 |
| 3 | original_firstorder_Skewness | 0.085255247 | 3 | original_firstorder_Kurtosis | 0.056335914 | 3 | pixel_spacing | 0.1646 |
| 4 | original_glcm_InverseVariance | 0.18798487 | 4 | original_firstorder_TotalEnergy | 0.257114485 | 4 | original_glcm_InverseVariance | 0.38651 |
| 5 | original_glcm_MaximumProbability | -0.21973734 | 5 | original_glcm_InverseVariance | -0.12113279 | 5 | original_shape_MajorAxisLength | 0.42426 |
| 6 | original_glszm_SizeZoneNonUniformityNormalized | -0.32487652 | 6 | original_glszm_SizeZoneNonUniformity | -0.5726098 | 6 | original_shape_LeastAxisLength | 0.26235 |
| 7 | original_glszm_GrayLevelVariance | -0.0201353 | 7 | original_glszm_SizeZoneNonUniformityNormalized | 0.150107862 | 7 | original_shape_Elongation | -0.4787 |
| 8 | original_glszm_ZoneEntropy | 0.083216907 | 8 | original_gldm_LargeDependenceEmphasis | 0.028058623 | 8 | Normalized_radial_lengths_mean | -0.0504 |
| 9 | original_shape_LeastAxisLength | 0.775575438 | 9 | original_gldm_SmallDependenceLowGrayLevelEmphasis | -0.02828542 | 9 | Area.ratio.of.macroscopic.contour | 0.3718 |
| 10 | original_shape_Elongation | -0.31950945 | 10 | original_shape_MajorAxisLength | 0.910837475 | 10 | textural_phenotype_level_80.90. | -0.3794 |
| 11 | original_shape_Flatness | -0.08291132 | 11 | original_shape_Elongation | -0.06072506 | 11 | PLBP_hist_tumor_orient2_8 | 0.2131 |
| 12 | Normalized_radial_lengths_entropy | -0.00298561 | 12 | Area.ratio.of.macroscopic.contour | 0.792747923 | 12 | PLBP_hist_tumor_orient4_1 | -0.4487 |
| 13 | Area.ratio.of.macroscopic.contour | 0.380855451 | 13 | textural_phenotype_level_0.10. | -0.0136375 | 13 | PLBP_hist_tumor_orient7_0 | 0.08372 |
| 14 | Roughness.index.of.boundary | 0.037077865 | 14 | textural_phenotype_level_M | -0.49383979 |  |  |  |
| 15 | textural_phenotype_level_20.30. | -0.02371688 | 15 | textural_phenotype_level_H | 0.18411398 |  |  |  |
| 16 | textural_phenotype_level_50.60. | 0.112047175 | 16 | PLBP_hist_tumor_orient0_9 | 0.039730751 |  |  |  |
| 17 | textural_phenotype_level_80.90. | -0.04633027 | 17 | PLBP_hist_tumor_orient1_0 | -0.81823034 |  |  |  |
| 18 | textural_phenotype_level_M | 0.012954773 | 18 | PLBP_hist_tumor_orient1_7 | -0.17617552 |  |  |  |
| 19 | PLBP_hist_tumor_orient0_7 | -0.1458134 | 19 | PLBP_hist_tumor_orient2_9 | 0.483604653 |  |  |  |
| 20 | PLBP_hist_tumor_orient5_0 | 0.165657527 | 20 | PLBP_hist_tumor_orient3_1 | 0.656039281 |  |  |  |
| 21 | PLBP_hist_tumor_orient6_7 | -0.35092263 | 21 | PLBP_hist_tumor_orient4_7 | -0.0295995 |  |  |  |
| 22 | PLBP_hist_tumor_orient7_0 | 0.09593962 | 22 | PLBP_hist_tumor_orient4_8 | 0.499052004 |  |  |  |
| 23 | WL_lbp_hist_cD1_7 | 0.101972769 | 23 | PLBP_hist_tumor_orient5_9 | 0.248384227 |  |  |  |
|  |  |  | 24 | PLBP_hist_tumor_orient6_7 | -0.20615164 |  |  |  |
|  |  |  | 25 | PLBP_hist_tumor_orient7_7 | -0.30720703 |  |  |  |
|  |  |  | 26 | WL_lbp_hist_cH2_1 | -0.03130493 |  |  |  |
|  |  |  | 27 | WL_lbp_hist_cH2_3 | 0.116690001 |  |  |  |
|  |  |  | 28 | WL_lbp_hist_cH2_6 | -0.01348323 |  |  |  |
|  |  |  | 29 | WL_lbp_hist_cH2_7 | 0.284480885 |  |  |  |
|  |  |  | 30 | WL_lbp_hist_cV2_1 | -0.28087589 |  |  |  |
|  |  |  | 31 | WL_lbp_hist_cV2_3 | 0.217091199 |  |  |  |
|  |  |  | 32 | WL_lbp_hist_cD2_1 | -0.04160241 |  |  |  |
|  |  |  | 33 | WL_lbp_hist_cH1_7 | -0.05733989 |  |  |  |
|  |  |  | 34 | WL_lbp_hist_cV1_1 | 0.094567049 |  |  |  |
|  |  |  | 35 | WL_lbp_hist_cD1_7 | -0.10230632 |  |  |  |
